# Applying 3D ED/MicroED workflows toward the next frontiers

**DOI:** 10.1107/S2053229624004078

**Published:** 2024-05-07

**Authors:** Mahira Aragon, Sarah E. J. Bowman, Chun-Hsing Chen, M. Jason de la Cruz, Daniel A. Decato, Edward T. Eng, Kristen M. Flatt, Sahil Gulati, Yuchen Li, Charles J. Lomba, Brandon Mercado, Jessalyn Miller, Lukáš Palatinus, William J. Rice, David Waterman, Christina M. Zimanyi

**Affiliations:** a Simons Electron Microscopy Center, New York Structural Biology Center, 89 Convent Ave, New York, New York 10027, USA; b Hauptman-Woodward Medical Research Institute, 700 Ellicott St, Buffalo, New York 14203, USA; c Department of Chemistry, University of North Carolina at Chapel Hill, Chapel Hill, North Carolina 27514, USA; d Memorial Sloan Kettering Cancer Center, 1275 York Avenue, New York, New York 10065, USA; e Chemistry and Biochemistry, University of Montana, 32 Campus Drive, Missoula, Montana 59812, USA; f Materials Research Laboratory, University of Illinois at Urbana Champaign, Urbana, Illinois 61801, USA; g Gatan Inc., Pleasanton, CA 94588, USA; h Department of Chemistry, Princeton University, Princeton, New Jersey 08544, USA; i Department of Physics, Quantitative Biology Institute, Yale University, 260 Whitney Ave., New Haven, Connecticut 06520-8103, USA; j Department of Chemistry, Yale University, New Haven, Connecticut 06520, USA; k Institute of Physics of the CAS/NanED, Na Slovance 1999/2, Prague 192000, Czech Republic; l Department of Cell Biology, NYU Grossman School of Medicine, 540 First Ave, New York, New York 10016, USA; m Research Complex at Harwell, UKRI–STFC Rutherford Appleton Laboratory, Harwell, Didcot, Oxfordshire, OX11 0FA, England, United Kingdom; University of Delaware, USA

**Keywords:** microcrystal electron diffraction, MicroED, 3D ED, electron diffraction

## Abstract

We report on the latest advancements in Microcrystal Electron Diffraction (3D ED/MicroED), as discussed during a symposium at the National Center for CryoEM Access and Training housed at the New York Structural Biology Center. This snapshot describes cutting-edge developments in various facets of the field and identifies potential avenues for continued progress. Key sections discuss instrumentation access, research applications for small mol­ecules and biomacromolecules, data collection hardware and software, data reduction software, and finally reporting and validation. 3D ED/MicroED is still early in its wide adoption by the structural science community with ample opportunities for expansion, growth, and innovation.

## Abstract 2

This topical review highlights recent 3D ED/MicroED developments and serves as proceedings of a symposium and workshop held on November 6–9, 2023, at the National Center for CryoEM Access and Training in New York, New York, USA.

## Introduction

Structural elucidation is a critical aim of many research groups involved in chemistry and structural biology. For decades, single-crystal X-ray diffraction (SCXRD) has been considered the ‘gold standard’ for high-resolution structural studies because of its well-established methodology. However, a significant barrier associated with SCXRD involves the growth of a suitable large crystal. This obstacle has driven the establishment of regional facilities and a national crystallization center for crystal growth (Lynch *et al.*, 2023 ▸). Similarly, reducing the need for large crystals (greater than 10 µm in all dimensions) (McPherson & Gavira, 2014 ▸) has motivated hard­ware developments. The advent of microfocus X-ray sources, liquid-metal-jet anodes, X-ray free electron lasers (XFEL), and improved detectors has allowed smaller and smaller crystals to be used for experimental studies. An alternative approach, taken by the structural biology field, is to forgo the crystal prerequisite and turn to imaging *via* CryoEM single-particle analysis (SPA) for mol­ecules over approximately 50 kDa in size (de la Cruz & Eng, 2023 ▸).

Developments in serial crystallography and XFEL techniques have pushed crystal-size requirements to new lows – although these methods require large qu­anti­ties of micro-sized crystals (Shoeman *et al.*, 2023 ▸). More recently, single-crystal electron diffraction methods have lowered the required size of crystals of all sources – macromolecular, small mol­ecules, materials, *etc*. – to smaller than the wavelength of visible light. The recent resurgence of three-dimensional (3D) electron crystallography methods has brought together chemists, biologists, theorists, programmers, crystallographers, and microscopists – a diversity reflected in a recent symposium held on November 6–9, 2023, at the National Center for CryoEM Access and Training (NCCAT) housed at the New York Structural Biology Center in New York City, USA. This review serves as a summary of the symposium. Our aim is to provide an overview of discussed topics that encourages collaboration, promotion, development, and expansion of 3D ED/MicroED.

### What’s in a name?

The NCCAT symposium included a roundtable discussion on the nomenclature of 3D electron crystallography, ack­now­ledging that various names used in the literature often differentiate data-collection methods (Saha *et al.*, 2022 ▸; Gemmi *et al.*, 2019 ▸). Although the methodologies may vary, the experimental outcome is the same: an atomic or near-atomic resolution model from measured diffraction intensities. The consensus at the symposium was that the semantics of the name should not distract from the goals of increasing access to this powerful technique by improving facility infra­structure, resources, and knowledge within the scientific community nor should it detract from the merits of a study. As a group, it was noted that choosing a single name for the technique would be useful from a literature search standpoint. Furthermore, it is well within the purview of the Inter­national Union of Crystallography (IUCr) to consider this matter in their electron crystallography discussions (*vide infra*). For this article, we will use the term 3D ED/MicroED to describe this technological approach.

## Instrumentation access today: bridging X-ray and CryoEM

Recording electron diffraction data for the purpose of 3D ED/MicroED requires a parallel beam of electrons, a stable sample stage, and a fast detector that can be used to both image microcrystals and record diffraction patterns. Most commonly, a transmission electron microscope (TEM) has been employed to con­duct these studies. Access to TEMs for 3D ED/MicroED varies widely in the research community, likely due to several reasons, including cost, a rapidly changing field, and high instrument demand. Access primarily falls into three categories: (1) academic core facilities, (2) com­mercial service providers, and (3) national resources.

Academic core microscopy facilities are often devoted to real-space imaging of macromolecules or inorganic materials to support the research goals of various departments. The detector advancements facilitating the ‘cryoEM resolution revolution’ also improved electron diffraction measurements and the quality of structures determined. However, pipelines for SPA CryoEM and a related technique, cryo-electron to­mog­ra­phy (CryoET), rapidly matured and, as a result, infra­structures at educational institutions and national resources have been prioritized to streamline high-value structure determination of biomacromolecules. This entanglement of TEM instrumentation with SPA CryoEM and CryoET of pro­tein targets may explain the slow growth of 3D ED/MicroED (particularly in the United States). Establishing 3D ED/MicroED workflows at academic core facilities often necessitates a cooperation between at least three parties: microscopist, crystallographer, and lab researcher. TEM facility managers and directors must also consider: (1) how the additional use will impact the availability of instrumentation to the core userbase and (2) the field of 3D ED/MicroED is still developing. Thousands of distributed crystalline grains can be inserted into the microscope at one time for analysis by depositing them on a TEM grid and sometimes only several of those will produce good-quality data to obtain the desired structure. Manual screening of single grains with asynchronous data reduction is currently the most common mode of operation. Automation tools for 3D ED/MicroED screening and data collection of entire grids are being developed and improved (Unge *et al.*, 2023 ▸; Hogan-Lamarre *et al.*, 2024 ▸). However, 3D ED/MicroED automation for sample screening and data collection is not widely available when compared to the resources for SPA CryoEM and CryoET. Further, the standardly available automated post-collection data processing pipelines that exist at X-ray crystallography beamlines have not been converted to use for electron diffraction sources. We are unaware of any com­mercially available options for automatic simultaneous mass screening and data reduction employing general purpose TEMs at the time of this writing. However, there are non-com­mercial solutions re­ported, such as *SerialRED* (Wang *et al.*, 2019 ▸), that pieces together *Instamatic* (Smeets *et al.*, 2021 ▸) with data reduction software *via* homegrown scripts to automatically collect and process large volumes of diffraction data.

The other two access points, com­mercial service providers and national facilities, frequently have experts in structure refinement on staff. Commercial service providers are typically for-profit groups that use privately held electron mi­cros­copy equipment for 3D ED/MicroED data collection. In contrast, national facilities offer publicly available microscopy to record diffraction data either as a service or under expert guidance of both microscopist and structural scientist. As one example, the recently established (2023) National Electron Diffraction Facility in the UK (https://www.ncs.ac.uk/nedf/) is dedicated to 3D ED/MicroED and does not employ TEMs but dedicated electron diffractometers.

Currently, 3D ED/MicroED instrumentation falls into one of two categories: (1) TEMs with minor changes to the hardware and software, and (2) purpose-built electron dif­frac­tom­eters based on TEM technology. Few purpose-built elec­tron diffractometers exist, with two vendors (Rigaku Corporation and Eldico Scientific AG) providing turn-key solutions for 3D ED/MicroED data collection. Both systems have collected high-quality diffraction data (Ito *et al.*, 2021 ▸; Simoncic *et al.*, 2023 ▸). These instruments could easily integrate into existing electron microscopy facilities. They are also ad­ver­tised to operate with higher tolerances of electromagnetic fields, vibration, and sound, which opens the possibility of these systems being installed in existing SCXRD laboratories.

**Figure 1:**
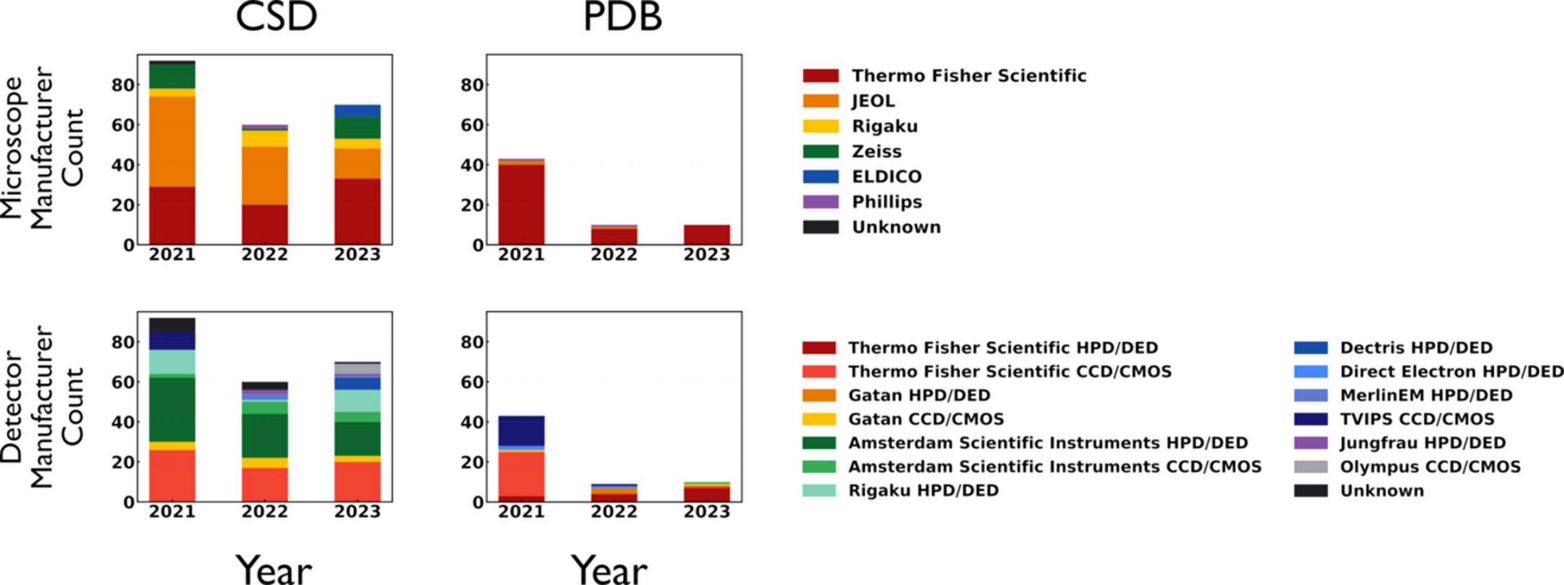
Histograms of 3D ED/MicroED structures within the PDB and CSD since 2021. The ‘/’ between detector styles (*e.g.* HPD/DED, CCD/CMOS) is intended to be read as ‘and/or.’ The grouping was chosen to simplify the bar color coding.

A search of the Protein Data Bank (PDB) and Cambridge Structural Database (CSD; Groom *et al.*, 2016 ▸) for recent structures determined by 3D ED/MicroED shows a trend previously described by Bruhn *et al.* (2021 ▸) that more small mol­ecule data is being generated than macromolecular data (Fig. 1 ▸). It is notable that in macromolecular work, Thermo Fisher Scientific (TFS) microscopes have been used for many of the published structures, whereas a split between Japan Electron Optics Laboratory Company (JEOL) and TFS in­struments have been used for the majority of small mol­ecule structures (Fig. 1 ▸). Another prominent difference is that macro­molecular diffraction patterns have been recorded mostly with direct electron detectors (DEDs) and hybrid pixel detectors (HPDs), whereas small mol­ecules are split between fiber-coupled complementary metal-oxide-semicon­ductor/charge coupled device (CMOS/CCD) detectors and DEDs/HPDs (Fig. 1 ▸). When correlated to instrument and detector type, recent de­po­si­tions show more diversity in collection hardware for small mol­ecules. Currently, the growth in small mol­ecule structure elucidation is set to outpace that of macro­molecular work paralleling X-ray data trends.

## Research applications of 3D ED/MicroED

### Small mol­ecules

**Figure 2:**
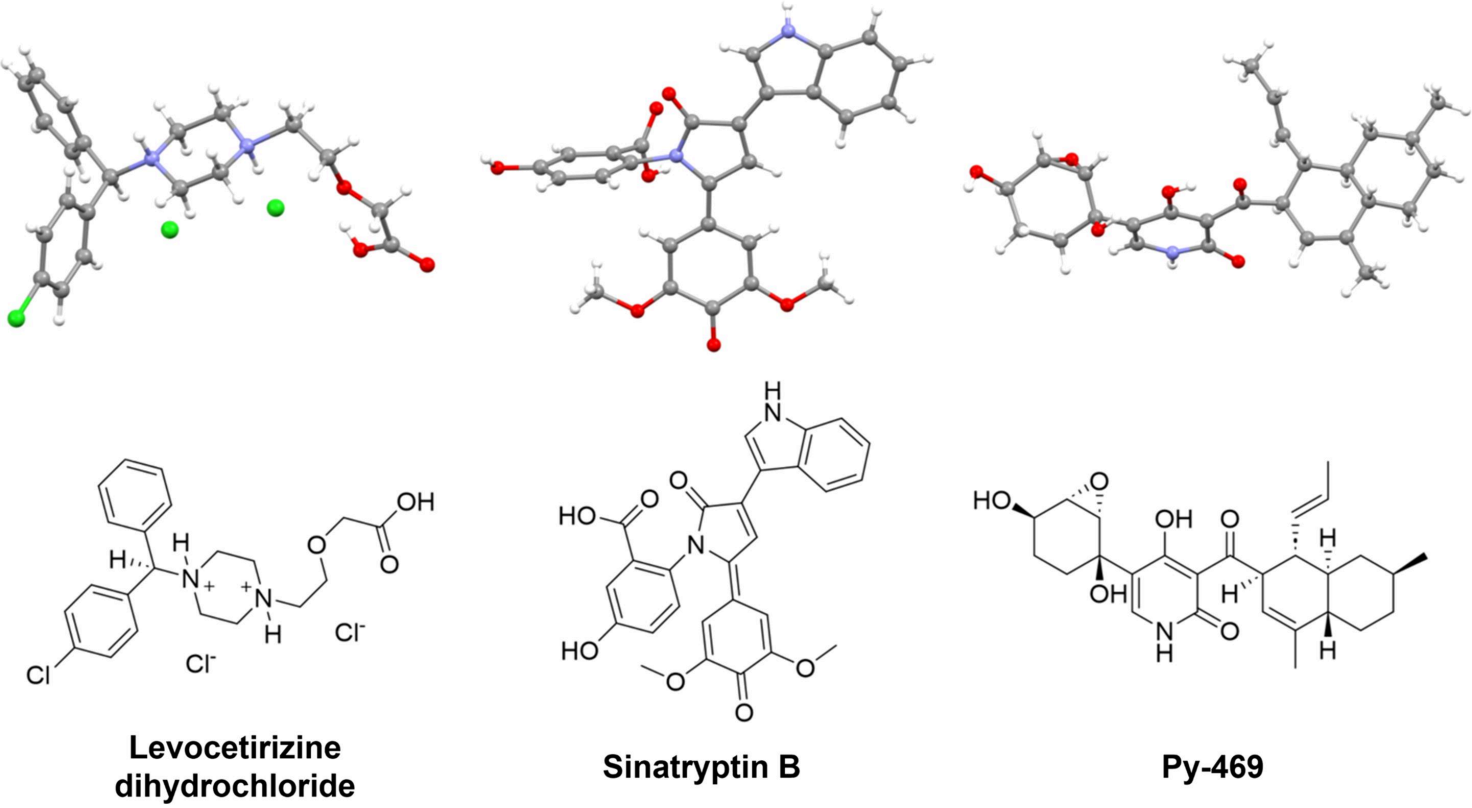
3D ED/MicroED structures of small mol­ecules from pharmaceutical and natural product research. Three specific examples of solved structures are shown in ball-and-stick rendering (top row) and as the chemical diagram (bottom row).

Some early small mol­ecule 3D ED/MicroED experiments on pharmaceuticals included reports showing the structures of carbamazepine, nicotinic acid, and paracetamol (Jones *et al.*, 2018 ▸; Gruene *et al.*, 2018 ▸). Scientists in the structure-based drug discovery space have continued to leverage 3D ED/MicroED, revealing the structures of several long-prescribed drugs, including mirabegron (Lin *et al.*, 2023 ▸), meclizine di­hydro­chloride (Lin *et al.*, 2024 ▸), and levocetirizine di­hydro­chloride (Fig. 2 ▸) (Karothu *et al.*, 2023 ▸) – a common anti­his­tamine drug that has been used for over 25 years. A timely example, given recent pharmaceutical industry headlines (Kingwell, 2023 ▸), was the structure determination of macrocycles from nanograms of material (Danelius *et al.*, 2023 ▸). The inherent difficulties (flexibility, solvent inclusion) associated with growing macrocycle crystals for SCXRD suggests 3D ED/MicroED will continue to play a role in macrocycle drug discovery.

Structure elucidation is a major part of the natural products workflow. Spectroscopic methods are often hindered by low yields and/or low proton content. Low yields can also limit the number of crystallization screens for traditional SCXRD studies. As such, 3D ED/MicroED has been used in several natural product elucidations, including the cytotoxic metabolite lomaiviticin C (Kim, Xue *et al.*, 2021 ▸), three algacidal metabolites sinatryptin B (Fig. 2 ▸), sinamicin B and C (Park *et al.*, 2022 ▸), as well as fungal metabolites Py-469 (Fig. 2 ▸) and fisherin (Kim, Ohashi *et al.*, 2021 ▸). It has also been leveraged to determine the relative configuration of an inter­mediate in the total synthesis of Securamine A, a cytotoxic alkaloid natural product isolated from marine invertebrates (Alexander *et al.*, 2024 ▸). A creative adaptation of 3D ED/MicroED to the natural products workflow merges microarray technology with on-grid crystallization (Delgadillo *et al.*, 2024 ▸). Nelson and co-workers have described the de­po­si­tion of picoliter-sized fractions of crude extracts from high-performance liquid chromatography directly onto a TEM grid. This permitted the time-resolved screening of 96 fractions on a single grid, demonstrating a new avenue for the high-throughput discovery of natural products. In a similar vein, 3D ED/MicroED has been envisioned as a component of the metabolomics analysis workflow (Ghosh *et al.*, 2021 ▸).

3D ED/MicroED has impacted areas of small mol­ecule chemistry beyond drug research. Time-resolved studies of car­bamazepine have evaluated the early stages of crystallization (Broadhurst *et al.*, 2021 ▸). Cocrystals from solid-state grinding provided structures inaccessible from solution-based crystallization (Sasaki *et al.*, 2023 ▸). Materials chemistry has benefited from 3D ED/MicroED growth as well, proving critical in the structure elucidation of metal–organic frameworks (MOFs) (Ge *et al.*, 2021 ▸, 2022 ▸), and covalent organic frameworks (COFs) (Zhou *et al.*, 2023 ▸). These crystalline solids are often used for a diverse range of applications – the performance of which is frequently attributed to the framework atomic structure. Framework synthesis frequently does not encourage the growth of large single crystals, often resulting in polycrystalline materials. Thus, structure elucidation often proceeds *in silico* or by powder XRD methods (Rietveld method). While MOFs are considered highly sen­si­tive to the electron beam, guidelines for data collection have been suggested (Yang *et al.*, 2022 ▸). Framework structure determination with 3D ED/MicroED is expected to become commonplace due to the symbiotic relationship between the small crystalline materials produced and the technique pre­re­quisite for vanishingly small crystals.

Lastly, one area that seems to be lacking at least in the USA is the application of the technique to inorganic materials. 3D ED/MicroED has been applied to alloys (Klementová *et al.*, 2017 ▸), epitaxial thin films (Steciuk *et al.*, 2019 ▸), and metal-ion battery sciences (Hadermann & Abakumov, 2019 ▸). In fact, a 2019 special edition issue on *Electron Crystallography* in *Acta Crystallographic Section B* (Hadermann & Palatinus, 2019 ▸) and a 2022 special edition on *Electron Diffraction and Structural Imaging* in *Symmetry* (Pratim Das *et al.*, 2022 ▸) highlight several structure elucidation examples with connections to solid-state chemistry and geology.

### Biomacromolecules

Many challenges in the crystallization of biomacromolecules for 3D ED/MicroED are the same as those for SCXRD. Finding conditions in which samples will form crystals is an ongoing bottleneck. Biomolecular crystals are delicate because their weak packing inter­actions result in large solvent content (typically 40–80%) and require significant care during handling. Some challenges, however, are unique. Mother liquors that maintain hydration around crystals inter­fere with electron transmittance and must be removed as much as possible without disrupting the solvent channels that help crystals maintain their packing. Many of the chemical com­ponents used for generating crystals are highly viscous, making them difficult to remove. Finally, because 3D ED/MicroED requires crystals smaller than the wavelengths of visible light, detecting potential crystallization conditions is a major bot­tle­neck. The question that arises most frequently is: ‘How do I know if I have micro/nanocrystals?’

A variety of solutions have been proposed to generate and detect biomolecular crystals of the appropriate size for 3D ED/MicroED. Often sample preparation involves manual manipulation of a crystal slurry (*via* sonication, crushing, or pipetting) to generate crystal fragments (de la Cruz *et al.*, 2017 ▸); this approach, however, can result in damage to the crystals, thus precluding good diffraction. An alternative approach has been developed that uses a cryogenic focused ion beam (cryo-FIB), in which ‘large’ (thicker than ∼1 µm) crystals are machined to the correct thickness for 3D ED/MicroED experiments (Martynowycz *et al.*, 2019 ▸; Duyvesteyn *et al.*, 2018 ▸). Cryo-FIB milling can also be used for specialized crystal growth methods, such as lipidic cubic phase (Martynowycz *et al.*, 2023 ▸; Polovinkin *et al.*, 2020 ▸). However, access/availability of cryo-FIB equipment is limited and varies across institutions. Additional advances have been made using negative stain TEM to identify microcrystals directly (Weiss *et al.*, 2021 ▸). Finally, approaches have been proposed that trans­late tools from X-ray crystallography to 3D ED/MicroED, including the use of non-linear optical (NLO) imaging to visualize biomolecular crystals already in the correct size regime (Li *et al.*, 2021 ▸; Miller *et al.*, 2022 ▸).

The other unique challenge for macromolecular crystals is transferring them in their mother liquors onto cryoEM grids that can be inserted into the TEM. Current methods make use of existing tools for cryoEM sample preparation: a pipette is used to transfer a few microliters of a crystal slurry to a grid, followed by filter paper blotting and plunging into liquid cryogens. This can be done using com­mercially available plunge freezing instruments or similar custom solutions, such as the Preassis method (Zhao *et al.*, 2021 ▸). Methods to avoid the use of pipetting have been published recently (Gillman *et al.*, 2023 ▸), but these samples have thus far been limited to the cryo-FIB milling pipeline. The continued development of new and reproducible sample preparation methods will benefit the field.

### Evaluating the suitability of 3D ED/MicroED

**Figure 3:**
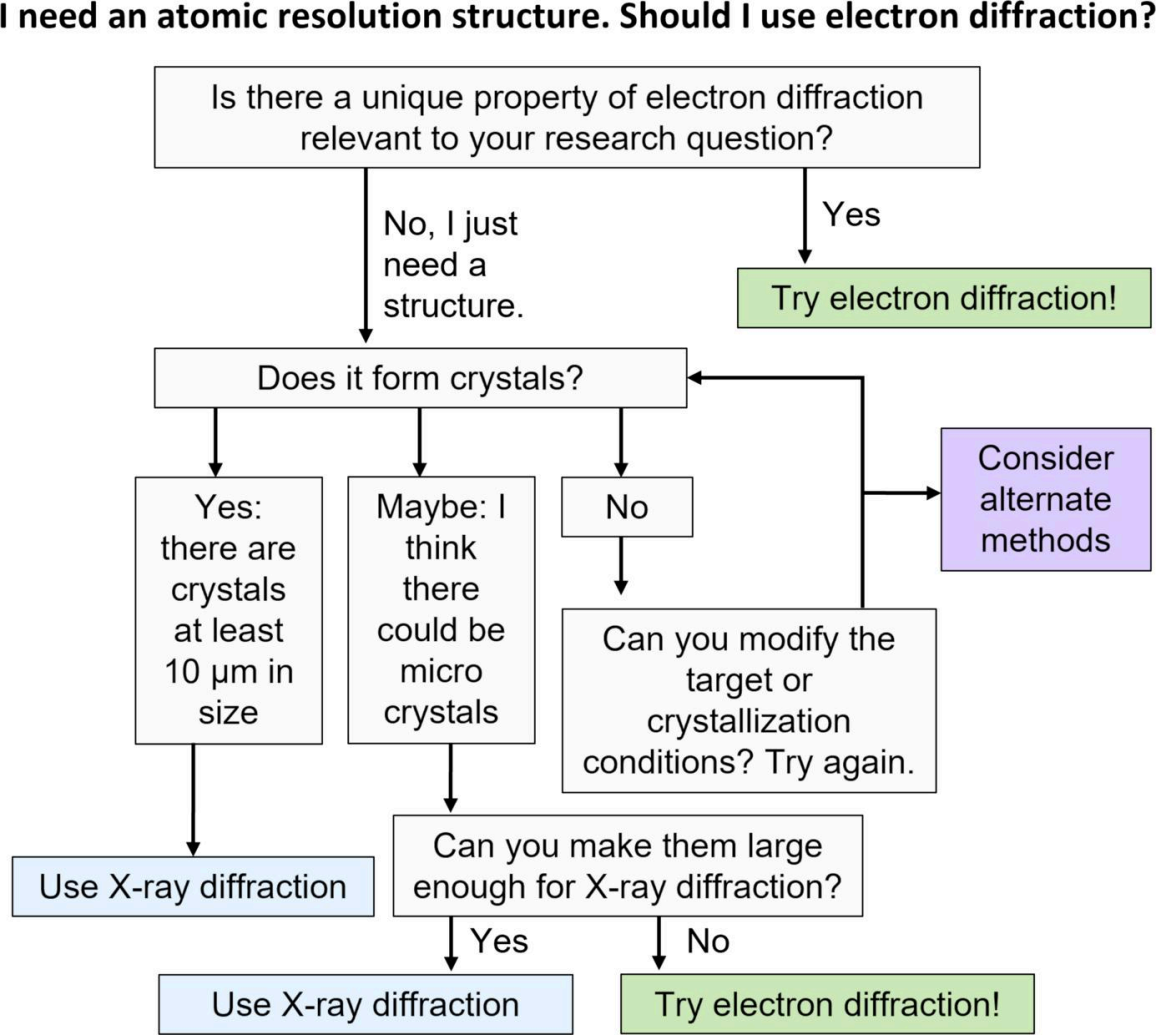
3D ED/MicroED decision tree. Just because you can use electron diffraction, doesn’t mean you must. Here we present a process to decide if 3D ED/MicroED is the most appropriate method for a structure elucidation project. Given the current state of the field, the use of more established X-ray techniques or alternative structure solution methods, when available, should be strongly considered as faster paths to useful data.

The power of 3D ED/MicroED lies in its versatility of samples. Theoretically, any analyte that forms crystals of appropriate size (too big can be an issue) that can diffract a weak electron beam is a candidate. However, the decision of whether to pursue this avenue over other techniques requires the consideration of several factors. As a starting point, we present a decision tree (Fig. 3 ▸) to aid researchers inter­ested in using this technique. While this decision tree does not encompass all potential circumstances scientists may en­coun­ter, such as considerations related to the amount of material, time investment, cost, and access to instrumentation and expertise, it serves as a guide to launch collaborative discussions. The tree we present is intended to complement a previously published decision tree focused on small mol­ecules (Ito *et al.*, 2021 ▸).

## Practical implementation of 3D ED/MicroED

### Data collection hardware and software developments

Technological advancements aimed at enhancing SPA CryoEM have also benefited 3D ED/MicroED. These im­provements include the integration of field emission gun emitters with narrower energy spread,^1^ autoloaders for sample changing efficiency, energy filters for removing inelastic scattering, and detectors with improved signal-to-noise ratio (SNR).

There are several scintillator-based fiber optic cameras ap­propriate for collecting electron diffraction data. These include ClearView, Rio, and OneView from Gatan, Ceta-D and Ceta-M from Thermo Fisher Scientific, and the XF series from Tietz Video and Image Processing Systems. These cameras can collect data of sufficient quality to solve structures and represent a basic solution for 3D ED/MicroED. However, scintillator-based fiber optic cameras introduce constraints on data quality due to the inherent noise that is introduced during the conversion of electrons to photons within the scintillator, *i.e.* the transmission of light through the fibers to the sensor, and subsequent frame readout processes. Addressing the inherent noise involves, in part, increasing the electron dose on the specimen to enhance the SNR. However, such an approach entails a trade-off, as it increases beam-induced damage to the specimen.

In contrast to scintillator-based fiber optic cameras, DEDs count individual electrons and markedly enhance the SNR. Electron counting eliminates signal read noise and variability from electron scattering, enhancing the detective quantum efficiency of the detector across all spatial frequencies. For 3D ED/MicroED, these detectors ensure precise measurement of Bragg intensities, particularly at high-resolution frequencies that are characterized by weaker intensities. The same DEDs that are utilized for high-resolution SPA CryoEM have been used to collect high-quality diffraction data in electron counting mode (Martynowycz *et al.*, 2023 ▸; Clabbers *et al.*, 2022 ▸). As reviewed recently (Hattne *et al.*, 2023 ▸), counting detectors improve the accuracy of the diffraction data, though care must be taken to minimize coincidence loss by keeping the electron flux as low as possible. In fact, for some fast direct detection systems, such as Gatan K3, Alpine, and Metro, a beamstop is no longer necessary if the electron flux is minimized.

Hybrid detectors represent a significant advancement in diffraction data collection, particularly for 3D ED/MicroED applications and offer several advantages. First, their high dynamic range enables the detection of both weak and strong signals with precision. Second, their ability to count individual electrons enhances the accuracy of Bragg intensity determination over the wide dynamic range. Third, they are radiation-hard, making them capable of withstanding high dose rates without being damaged. Last, hybrid pixel detectors are fast which allows them to capture diffraction data quickly. Due to their pixel design characteristics, hybrid pixel detectors typically feature larger pixel sizes compared to imaging detectors, resulting in fewer pixels on the chip. Common configurations include 256 × 256, 512 × 512, or 512 × 1024 pixel arrays. These detectors are offered by various manufacturers, such as Gatan,^2^ Dectris, Amsterdam Scientific Instruments, Quantum Detectors, Rigaku, and X-spectrum, providing a diverse range of options.

A variety of data collection software packages are com­mercially available, often optimized for specific detectors, including *Latitude D* from Gatan, *EPU-D* from TFS, and other packages for dedicated instruments. Additionally, open source packages are available, including *Leginon* (Cheng *et al.*, 2021 ▸), *Instamatic* (Smeets *et al.*, 2021 ▸), *SerialEM* (de la Cruz *et al.*, 2021 ▸), and *ParallelEM* (for JEOL microscopes) (Yonekura *et al.*, 2019 ▸). All the options save data in formats compatible with existing data reduction software, however, challenges may arise in accurately importing the necessary metadata. It is therefore recommended to con­duct preliminary tests to establish an optimal workflow from data collection to structure solution whenever using new data collection software for the first time.

Specialized instrumentation dedicated to electron diffraction based on TEM technology have also emerged, including systems developed by Rigaku Corporation (XtaLab Synergy-ED) and Eldico Scientific AG (ED-1). These electron diffractometers are optimized for small mol­ecule analysis rather than frozen-hydrated protein specimens, although they offer cryogenic cooling capabilities as an optional feature. Operating at up to 200 keV (ED-1 reported at 160 keV) and equipped with LaB_6_ filaments, these systems offer streamlined designs and functionalities. They feature built-in hybrid de­tec­tors and dedicated data collection software. Rigaku’s system is built on existing technology from JEOL, leveraging a JEM-2100 framework paired with *CrysAlis PRO* software that facilitates simultaneous unit-cell reduction and data integration. The ED-1 features a horizontal beam path reminiscent of X-ray diffractometers, no post-sample lenses, and a unique five-axis translational goniometer. Eldico’s software provides immediate feedback on crystallographic parameters, but ultimately data are exported for data reduction using external crystallographic software packages.

### Data reduction software and processing

Early electron diffraction experiments utilized geometries distinct from the standard Arndt–Wonacott rotation method employed widely in X-ray experiments. Precession electron diffraction (Midgley & Eggeman, 2015 ▸; Vincent & Midgley, 1994 ▸) is one such geometry, used to integrate through the Bragg condition for reflections in a zone axis orientation, in a way that considerably reduces the effect of dynamic diffraction. Limitations of electron microscope tilt stages meant that early analogues of the rotation method were limited to discrete tilts, as with the automated diffraction to­mog­ra­phy method (Kolb *et al.*, 2008 ▸), or combinations of coarse stage tilts with fine beam rotations, as in the rotation electron diffraction (RED) method (Wan *et al.*, 2013 ▸). The diverse experiment geometries meant that software for electron diffraction data processing was developed independently from X-ray data processing software. Packages such as *ADT3D* (Kolb *et al.*, 2011 ▸), *RED* (Wan *et al.*, 2013 ▸), and *PETS2* (Palatinus *et al.*, 2019 ▸) are specialized for electron diffraction experiments with particular collection geometries.

Improvements in hardware and data collection methodologies have allowed convergence between electron and SCXRD experiments. The widely adopted continuous rotation data collection method is essentially identical in the abstract but exhibits distinct practical limitations governed by instrument properties. This convergence has allowed software originally written for SCXRD to be adapted for use in electron diffraction experiments, including widely used *SAINT* (Bruker) and *CrysAlis PRO* (Rigaku OD). *MOSFLM* (Battye *et al.*, 2011 ▸) was used particularly for early experiments with discrete tilts or wide-sliced rotation images. As for SCXRD, fast low-noise detectors allow for fine-sliced experiments, often processed by *XDS* (X-ray Detector Software) (Kabsch, 2010 ▸) or *DIALS* (Diffraction Integration for Advanced Light Sources) (Winter *et al.*, 2018 ▸). In addition, electron diffraction-specific software, such as *PETS2*, has been optimized for use with continuous rotation data. The adoption of software de­veloped for X-ray crystallography in 3D ED/MicroED brings benefits, such as empirical profile modelling (standard in SCXRD for decades), as well as familiarity and support from a wide userbase.

*DIALS* stands apart from the other SCXRD processing packages in that it has not merely been borrowed for electron diffraction. Since 2018, various features have been added to the package to specifically support 3D ED/MicroED. An initial publication (Clabbers *et al.*, 2018 ▸) detailed some of these, including distortion correction maps, modelling of beam drift, and diagnostics for geometry refinement. Since then, electron-diffraction-focused development has continued, bringing improved spot-finding methods for integrating de­tec­tors like the Ceta-D, rotation axis determination using the algorithm of Kolb *et al.* (2009 ▸), and image format readers for a wide variety of instruments and detectors.

The issue of file formats for 3D ED/MicroED data remains a major obstacle to progress in the field. Standard electron microscopy formats are geared towards imaging applications and lack sufficient metadata to describe diffraction experiments. This has led many labs to develop homegrown file format conversion pipelines. As lamented previously (Water­man *et al.*, 2023 ▸), the chosen output format is often deficient in some way, leading to problems, such as incomplete metadata, poor compatibility between programs, and the potential for structural misinter­pretation, as in the case of images with a flipped axes. The proposal for a standardized format based on best practice taken from the X-ray macromolecular crystallography community is an important one (Waterman *et al.*, 2023 ▸). At present, however, use of the proposed format has only been demonstrated at a single site, namely, eBIC at Diamond Light Source. Unlocking the potential for 3D ED/MicroED to achieve high-throughput automated data pro­cessing at any lab, using a standardized data format, will require the community to come together and apply pressure on instrument manufacturers to support such a standard format from the point of data acquisition.

### Data reporting and validation

#### Small mol­ecule

A notable aspect of crystallography as a science and a technique is the well-developed and elaborate system of universally adopted standards for data reporting and validation. An initiative under the auspices of Inter­national Union of Crystallography (IUCr) started in 1990s with the aim of providing a standard for sharing crystallographic data. The result was a file format named Crystallographic Information File (CIF), later extended beyond a simple file format, and changed to Crystallographic Information Framework, with the acronym CIF standing for both these expressions. The CIF standard has been designed to permit encoding of all important information related to the deposited crystal structure. An important tool related to the CIF standard is the *checkCIF* software (Spek, 2020 ▸). This set of tools developed by Ton Spek performs a thorough validation of the structure in the CIF both in terms of syntactic correctness and – even more importantly – agreement of the deposited structure with standard quality requirements and crystallochemical expectations.

Essentially, all crystal structures published currently are deposited as a CIF file and subject to the *checkCIF* procedure. However, the CIF format and *checkCIF* software were both designed before the advent of 3D ED/MicroED. Given the sometimes significant differences between X-ray and electron diffraction, relevant questions may be asked: is the CIF a suitable format for the de­po­si­tion of 3D ED/MicroED data? Is *checkCIF* a useful tool for validating 3D ED/MicroED structures?

The simple answer to both these questions is ‘yes’. How­ever, a few particularities of 3D ED/MicroED structures require specific attention. First, structures obtained by kinematical and dynamical refinement must be distinguished. The kinematical case is closer to the standard X-ray case; for ex­ample, symmetry-equivalent reflections are merged and averaged, and the structures are refined using the same parameters as X-ray structures. This treatment means that the structure can be reported in the CIF format without any issue – all necessary keywords are available. However, the figures of merit (*R*_int_, *R*_obs_, *etc*.) are significantly higher than the typical values for SCXRD structures (*R* factor ≃ 4–6% for small mol­ecule organic SCXRD structures *versus* ≃ 15–20% for kinematical refinement of 3D ED/MicroED structures), and the accuracy of the refined structures tends to be lower than the standard expected by *checkCIF*. As a result, several alerts of type A (potentially serious errors) and B (potentially significant problems) are often issued by *checkCIF*. These alerts include alerts on elevated figures of merit or alerts on a too-low C—C bond precision. While these alerts can be easily explained by the fact that the structure is a 3D ED/MicroED structure, it also means that the validation performed by these alerts is no longer effective, and ignoring these alerts might lead to overlooking valid problems. A possible remedy could be modifying *checkCIF* so that the thresholds for issuing alerts and classifying them as A or B would be relaxed to some extent for 3D ED/MicroED structures.

The second case is dynamical refinement. The dynamical refinement is distinct from the kinematical one in several ways. Most notably, it employs dynamical diffraction theory, which leads, in general, to lower figures of merit and better accuracy of the refined structures. As a result, *checkCIF* alerts, due to the high figures of merit and low accuracy, are less frequent with dynamical refinement. On the other hand, dynamical refinement does not employ reflection merging, because dynamical effects are different for each of the symmetry-equivalent reflections. Moreover, reflection scaling cannot be performed during the data reduction stage but must be part of the refinement process. As a result, the *F*_o_–*F*_c_ list is not compatible with *checkCIF*, and the part of *checkCIF* that processes the reflection list cannot be used for dynamical refinement. Unlike kinematical refinement, dynamical refinement permits the determination of absolute structure and absolute configuration. However, the method used is different from the anomalous dispersion effects exploited in X-ray crystallography (Brázda *et al.*, 2019 ▸; Klar *et al.*, 2023 ▸). The CIF standard does not provide suitable options to specify the absolute structure determination results by dynamical refinement, and, for the time being, this information needs to be specified within the free text of the keyword _refine_special_details.

The issues with CIF and *checkCIF* for kinematical and dynamical refinements are the subject of debate by the Standardization Com­mittee of a European Union MSCA (Marie–Sklodowska–Curie–Action) project titled NanED (Electron Nanocrystallogrpahy) (https://naned.eu/). One of the goals of NanED is to propose improvements to the CIF standard and *checkCIF* procedure to also be suitable for 3D ED/MicroED structures. The suggestions formulated by the standardization committee, after discussing with the broad electron-crystallography community, will be proposed to the IUCr’s Com­mittee for the Maintenance of the CIF Standard and, hopefully, adopted as an update of the CIF standard. Thus, within a few years, the remaining issues with CIF and *checkCIF* for 3D ED/MicroED should be resolved, and re­porting 3D ED/MicroED structures should become as simple and as standardized as those obtained by X-ray or neutron diffraction methods.

#### Biomacromolecule

The PDB is adapting to accommodate the de­po­si­tion of biomacromolecular structures solved using electron diffraction. Which was the best flag for 3D ED/MicroED was not standardized early on, making it difficult to sort and find 3D ED/MicroED data specifically. The PDB recognizes that the current handling of the data makes it difficult for users to find 3D ED/MicroED structures and are proactively taking action to address the issue. They are extending the PDBx/mmCIF dictionary to meet feedback already gathered from the broader electron crystallography community. These include providing a tag to distinguish 3D ED/MicroED from 2D electron crystallography or 3DEM (three-dimensional electron microscopy), changing the de­po­si­tion requirements for experimental data (*i.e.* making the de­po­si­tion of structure factors mandatory), extending the data model for metadata unique to 3D ED/MicroED, and defining additional mandatory metadata relevant to 3D ED/MicroED. In brief, they will offer example files and an extended dic­tio­nary to the wwPDB GitHub for community feedback in an iterative manner. The PDB is actively working on these enhancements and welcomes contributions from those inter­ested in supporting their efforts – those who are inter­ested in contributing to the efforts should contact the PDB.

## The future of 3D ED/MicroED

3D ED/MicroED has broadened the scope of structural sciences by partially addressing the bottleneck of crystal growth. The challenge of crystal growth, however, will remain a critical factor due to the inherent nature of the technique. As such, crystal identification, crystal growth methods, and sample preparation (particularly for macromolecular species) require continued innovations.

For small mol­ecules, the inclusion of powder X-ray diffraction is a useful tool to pre-screen samples for 3D ED/MicroED. A powder pattern demonstrates sample crystallinity and suggests a high probability of success before loading it into the instrument. This quick screen helps justify the downstream costs and time investment required for 3D ED/MicroED structure elucidation. Furthermore, it would help managers ensure that instruments of high demand are being used efficiently.

As discussed previously, biomacromolecular crystals are sensitive to physical manipulation, but they can also exhibit a preferred orientation on cryoEM grids, making it difficult to get complete data sets. While also a problem for small mol­ecule data sets, this is a more significant problem for macromolecular studies, as the resolution is typically lower. To mitigate preferred orientation, researchers have leveraged 3D printing to create suspension drop vapor diffusion caps that hold TEM grids. This enables crystallization directly on the grid allowing researchers to omit transfer steps entirely and alleviate preferred orientation (Gillman *et al.*, 2023 ▸). Expansion of on-grid crystallization in combination with the microarray technologies described above highlight a possible future of on-grid crystallization screens akin to ‘setting up trays’, a possible natural progression for macromolecular 3D ED/MicroED.

Macromolecular samples, as well as some solvent-dependent small mol­ecule crystals, sometimes suffer from inadequate sample preparation (*i.e.* not enough blotting, resulting in a vitrified or frozen solvent too thick for 3D ED/MicroED). FIB-milling thick samples has proven effective for 3D ED/MicroED sample preparation, yet radiation damage and low sample throughput have necessitated further improvements to the workflow (Martynowycz & Gonen, 2021 ▸). Plasma-FIBs are a potential solution, as plasma sources offer a higher through­put with less radiation damage specifically for biological samples (Martynowycz *et al.*, 2023 ▸). Another sample pre­paration approach may be to mimic SPA automated sam­ple preparation routines, such as Spotiton (Dandey *et al.*, 2018 ▸), to deposit solutions of biological microcrystals to minimize the risk of vitrified ice being too thick and to enhance reproducibility.

A unique facet of 3D ED/MicroED is the rapidity with which data can be collected, in many cases taking less than one minute per crystal. This process, coupled with the potential for thousands of crystals on a grid, demonstrates that true automation will be a necessity to unlock the potential of the method. 3D ED/MicroED can take inspiration from SPA workflows, pipelines, and technology. For example, in the SPA workflow, users can select multiple areas of a grid for data collection without screening each individual point beforehand, which drastically reduces the amount of time required to set up an experiment. Data can then be processed on-the-fly. A ‘live’ software solution for 3D ED/MicroED that can identify crystals, process unit cells, and identify which data sets have a high probability of obtaining a structure would greatly im­prove the workflow. In fact, homemade solutions have been reported by a couple of groups, but widespread adoption has yet to be realized. *SerialRED* is one early example leveraging *Instamatic* with data-reduction software (Wang *et al.*, 2019 ▸). Another recent example paralleling this approach was automated data collection with *SerialEM* coupled with homemade scripts for data reduction (Unge *et al.*, 2023 ▸). Both examples foreshadow a possible route to automated workflows. Overall, streamlined automation and user-friendly solutions addressing bottlenecks of data acquisition and data processing would make 3D ED/MicroED more routine and enable creative new approaches in the structural sciences.

## Conclusion

Researchers who venture into 3D ED/MicroED quickly note a dynamic field propelled by a diverse community of dedicated chemists, biologists, theorists, programmers, crystallographers, and microscopists. This diversity was evident in the attendance at the workshop and symposium at NCCAT. To support the rapidly developing 3D ED/MicroED ecosystem, further de­vel­op­ment is needed in several areas.

**Figure 4:**
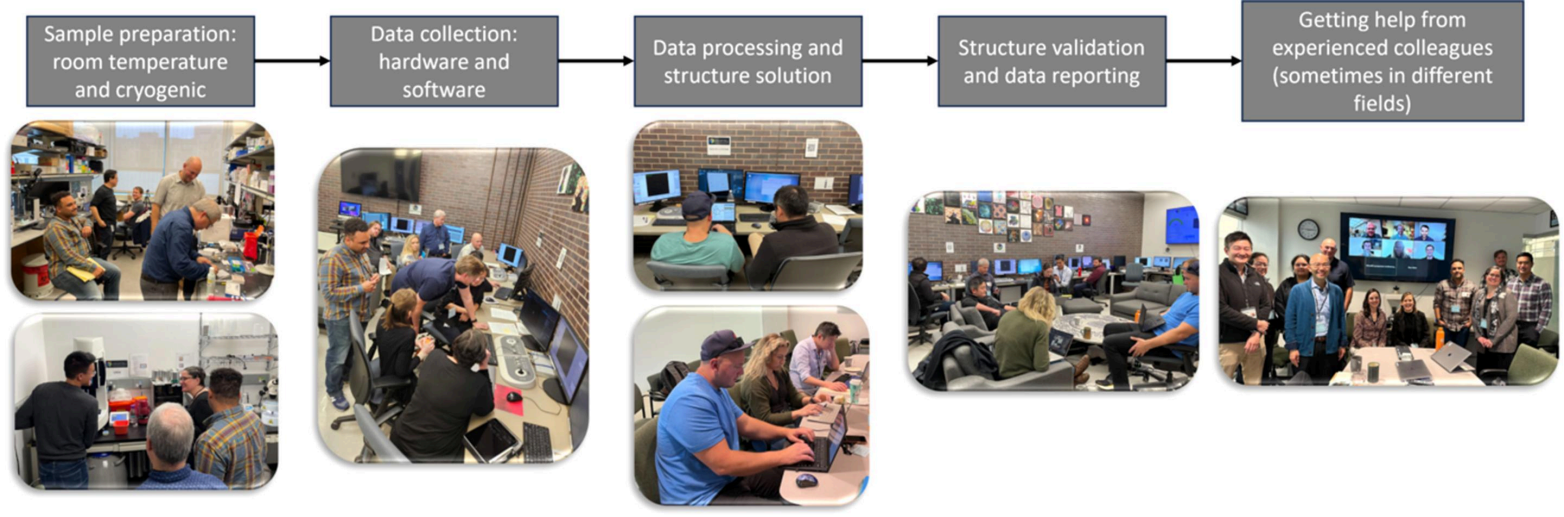
Exploring practical challenges in 3D ED/MicroED: highlights from the workshop.

One area that demands additional fostering is the expansion and support of 3D ED/MicroED workshops and symposia. Dis­cussions of, ‘How do ***you*** do it?’, in person are critically beneficial to the participants (and organizers!). The value of hands-on experience and conversations with colleagues pos­sessing more expertise cannot be understated (Fig. 4 ▸). Along these lines it is critical to establish and continue to nurture partnerships with technology developers from both the public and private sectors to ensure barriers lowered by the technology are not replaced with new logistical barriers (*e.g.* access, cost, expertise, sample prep, data analysis, *etc*.). Archival and reporting teams (IUCr, NanED, PDB, Cam­bridge Crystallographic Data Centre) are setting the standard by taking meaningful actions in response to community feedback. Similar activity should be expected from the private sector; end users are encouraged to express the need for standard image formatting to microscope and detector com­panies. Actions here will improve the workflow and per­mit those involved in con­ducting 3D ED/MicroED studies to concentrate on impactful research in place of data formatting woes.

A second area of development concerns access to 3D ED/MicroED instrumentation. Currently there appear to be two distinct groups in United States academia. One front consists of labs who have capable equipment (*e.g.* core facilities focused on SPA and to­mog­ra­phy) but lack extensive experience. These labs are expanding capabilities by establishing 3D ED/MicroED workflows on existing multi-purpose instruments and publishing data sporadically. Another group of labs are focused on technique development, and work on the bleeding edge of what is possible. With dedicated instrumentation and extensive expertise, these groups can redefine approaches to answer research questions and publish frequent exciting results. The notable achievements of these technology development groups often overshadow the variable success rate and the challenges associated with 3D ED/MicroED experiments at facilities that are integrating diffraction data collection as one of many services available to users. In contrast, 3D ED/MicroED in Europe is primarily being con­ducted in crystallographic core facilities. This presents two different approaches that have evolved based largely on institutional organization and funding differences.

To address access needs, variable success rates, and general 3D ED/MicroED challenges, a diversified approach seems logical. Expanding the adoption of 3D ED/MicroED across campus core facility requires collaborative efforts and a time investment by multiple parties, sometimes spanning different departments or universities. This separation of inter­ested parties justifies the need for workshops/symposia to facilitate rapid and efficient communication. Another point to note is that costs can be expensive ($1k–3k/day for a local facility) for smaller labs thereby inhibiting access to some. Establishment of national labs or national services could lower the burden of oversubscribed core facilities and provide access to smaller research labs or researchers without localized infra­structure. National diffraction labs could operate and provide expertise similar to synchrotrons and CryoEM facilities. At such facilities, there is incentive for the development of automation and user-friendly solutions addressing the full range of bottlenecks in 3D ED/MicroED, from sample preparation to data acquisition and processing. There is potential at the three national CryoEM facilities in the USA to step into this role. While they have some inter­est in MicroED, none of them officially offer these services in the same way that they support SPA CryoEM – however they are limited by what funding dictates they focus on. Lastly, continued support of individual labs both developing and using the methods needs to be protected as they have and will continue to play a critical role in driving innovation.

3D ED/MicroED has proven to be a valuable technique for structure elucidation, showcasing substantial opportunities for expansion, growth, and innovation. Navigating the evolving landscape of this developing technique presents both challenges and exciting possibilities, providing us with a unique opportunity to witness history and a potential boom in structural science. The promising future of 3D ED/MicroED should evoke excitement for all in our field.

## Conflict of inter­est statement

SG is an employee of Gatan Inc., which developed and is marketing the Alpine, Metro, K3, ClearView, Rio, and OneView cameras, and *Latitude D* software.

## Supplementary Material

Refcodes used in the construction of the histograms in Fig. 1. DOI: 10.1107/S2053229624004078/yp3235sup1.pdf

## Footnotes

1 A FEG source will give you a more coherent beam and thus more diffraction signal at lower total dose. Likely it is not important for small mol­ecules, but it is for protein crystals, where you need all the signal you can get from the small number of unit cells and it is likely beneficial. Furthermore, with protein crystals, often the high-resolution signal rapidly disappears with radiation damage so you can’t just increase the brightness (*i.e.* increase dose).

2 Stela camera utilizes the DECTRIS hybrid-pixel electron detector that employs electron counting to minimize noise.
