# Supplementary material for: Applying 3D ED/MicroED workflows toward the next frontiers
Source: Acta Crystallogr C Struct Chem. 2024 May 7;80(Pt 6):179–89. doi: 10.1107/S2053229624004078 (PMC11150879; doi:10.1107/S2053229624004078)
Supplement: Supplementary file 1 [file c-80-00179-sup1.pdf]

Acta Cryst C

STRUCTURAL CHEMISTRY

Volume 80 (2024)

Supporting information for article:

Applying 3D ED/MicroED workflows toward the next frontiers

Mahira Aragon, Sarah E. J. Bowman, Chun-Hsing Chen, M. Jason de la Cruz, Daniel A. Decato, Edward T. Eng, Kristen M. Flatt, Sahil Gulati, Yuchen Li, Charles J. Lomba, Brandon Mercado, Jessalyn Miller, Lukáš Palatinus, William J. Rice, David Waterman and Christina M. Zimanyi

CSD Refcodes for construction of Fig. 1.

2023

2160709	2169344	2242895	2219079	2218795	2260059	2246164	2242893
2192858	2169349	2246160	2219080	2237143	2263467	2168647	2242894
2192859	2172884	2246161	2219081	2246152	2268164	2259807	2169342
2192980	2172895	2174023	2225208	2246153	2268165	2259814	2169343
2222591	2172899	2192026	2225209	2246154	2268166	2206064	2246158
2238735	2268169	2192027	2233879	2246155	2268167	2211429	2246159
2239391	2268170	2192028	2238807	2246156	2268168	2167934	2245942
2242890	2268171	2201747	2243533	2246157	2169341	2205804	2254175
2242891	2268172	2201748	2242892	2268173	2201749		

2022

2141823	2177984	2124897	2183351	2095905	2123646	2217450	2123643
2141825	2194031	2124898	2183352	2095958	2123647	2182819	2123644
2144129	2208429	2126160	2183353	2107470	2124118	2119570	2069514
2144130	2208444	2126161	2183354	2118442	2124119	2120870	2090060
2147971	2265251	2127767	2190211	2118443	2107470	2167919	2167934
2148006	2101716	2141822	2190212	2119504	2130868	2168148	2178208
2149680	2111213	2124120	2193439	2119505	2132512	2152961	2121221
2152260	2114987	2162986	2212820				

2021

2112212	2073254	2097605	2087930	2026247	2084732	2063944	2179900
2112211	2040889	2097603	2087929	2026244	2063953	2063943	2083844
2112210	2013738	2097602	2087928	2020510	2063952	2061568	2238735
2112209	1979160	2097601	2084744	2015361	2063951	2061567	2086493
2097606	1914225	2097597	2038336	2150138	2063950	2061566	2150135
2097604	2112208	2097596	2038335	2150137	2063949	2061565	2097600
2087354	2112207	2096328	2038334	2150136	2063948	2046826	2097594
2080221	2112206	2091280	2026252	2097629	2063947	2040884	2097595
2080220	2112205	2091277	2026251	2097599	2063946	2038723	2085760
2073255	2103784	2087931	2026249	2087927	2063945	2032840	2085761
2020516	2039244	2085759	2096745	2102279	2179901	2179899	2062671
2023659	2061437	2033973	2033974				

PDB Ref Codes for construction of Figure 1.

2023

8CPC	8FYQ	8IJZ
8FYN	8FYR	8SKW
8FYO	8FYS	8SDK
8YFO		

2022

7ULY	8E53	8EUN
7UTE	8E54	8DDG
7XKJ	8EUM	8E52

2021

7MRP	7RVC	7SW1
7N2D	7RVD	7SW2
7N2E	7RVE	7SW3
7N2F	7RVF	7SW4
7N2G	7RVG	7SW5
7N2I	7RVH	7SW6
7N2J	7RVI	7SW7
7N2K	7RVJ	7SW8
7N2L	7RVK	7SW9
7QGQ	7RVL	7SWA
7RM5	7SKW	7SWB
7SVZ	7SKX	7SWC
7SW0	7SVY	7SXN
7VI4	7VI5	7T3H
